# The transcription factor MafB promotes anti-inflammatory M2 polarization and cholesterol efflux in macrophages

**DOI:** 10.1038/s41598-017-07381-8

**Published:** 2017-08-08

**Authors:** Hwijin Kim

**Affiliations:** 1 Center for Computational and Integrative Biology, Massachusetts General Hospital, Boston, MA 02114 USA; 2 Department of Genetics, Harvard Medical School, Boston, MA 02115 USA

## Abstract

Macrophages play pivotal roles in the progression and regression of atherosclerosis. Accumulating evidence suggests that macrophage polarization into an anti-inflammatory M2 state is a key characteristic of atherosclerotic plaques undergoing regression. However, the molecular mechanisms underlying this potential association of the M2 polarization with atherosclerosis regression remain poorly understood. Further, human genetic factors that facilitate these anti-atherogenic processes remain largely unknown. We report that the transcription factor MafB plays pivotal roles in promoting macrophage M2 polarization. Further, MafB promotes cholesterol efflux from macrophage foam cells by directly up-regulating its key cellular mediators. Notably, MafB expression is significantly up-regulated in response to various metabolic and immunological stimuli that promote macrophage M2 polarization or cholesterol efflux, and thereby MafB mediates their beneficial effects, in both liver x receptor (LXR)-dependent and independent manners. In contrast, MafB is strongly down-regulated upon elevated pro-inflammatory signaling or by pro-inflammatory and pro-atherogenic microRNAs, miR-155 and miR-33. Using an integrative systems biology approach, we also revealed that M2 polarization and cholesterol efflux do not necessarily represent inter-dependent events, but MafB is broadly involved in both the processes. These findings highlight physiological protective roles that MafB may play against atherosclerosis progression.

## Introduction

Atherosclerosis is characterized by excess accumulation of cholesterol-rich, apolipoprotein B (apoB)-containing lipoprotein in the subendothelial space (intima) of susceptible areas of arteries, which in turn triggers chronic inflammatory responses^1^. The recruitment of monocyte-derived cells into the subendothelial space, where they differentiate into macrophages and then ingest the accumulated lipoproteins to become the cholesterol-laden foam cells, is the key (patho-)physiological inflammatory process underlying atherosclerotic plaque development. Accumulating evidence indicates that macrophages in atherosclerotic lesions comprise heterogeneous cell populations that adapt their functional phenotypes in response to specific microenvironmental signals, which in turn plays key roles not only in atherosclerotic plaque progression but also in its regression^2^. Specifically, phenotypic shift of plaque macrophages, from a classically-activated and pro-inflammatory (“M1”) state to an alternatively-activated and anti-inflammatory (“M2”) state, appears to play important roles in promoting atherosclerotic plaque regression.

Reverse cholesterol transport (RCT) is a pathway by which excess cholesterol accumulated in peripheral tissues is delivered to the liver, which then can be removed from the body via secretion into the bile and subsequent disposal via the feces^3^. High-density lipoprotein (HDL) cholesterol is believed to play a key role in promoting the efflux of excess cholesterol from macrophage foam cells. Further, liver x receptor (LXR) has been shown to play a critical role in RCT by up-regulating expression of several genes that promote cellular cholesterol efflux, including ATP-binding cassette transporters Abca1 and Abcg1, and apolipoprotein E (Apoe), which in turn promoted atherosclerotic plaque regression in mouse models^4^.

We previously identified the transcription factor MafB as a negative regulator of cellular induction of type I interferon (IFN) and a subset of pro-inflammatory cytokines, protecting against the development of auto-inflammatory disorders in multiple mammalian tissues^5^. MafB belongs to the large Maf family of transcription factors, which consists of MafB, c-Maf, MafA and NrL. Among these, MafB, and to a lesser extent c-Maf, is expressed at an especially high amounts in myeloid cells of the hematopoietic system, and facilitates the establishment and maintenance of the monocyte-macrophage lineage^6^. Based on these previously findings, we explored the possibility that MafB may have even broader functional roles in inflammatory responses and M1/M2 polarization of macrophages. Further, we examined whether MafB plays a physiological functional role in regulating macrophage cholesterol efflux and thus the development of atherosclerosis.

We report here that MafB acts to drive macrophage phenotype into a less pro-inflammatory (M2) state by directly up-regulating expression of M2 marker genes while suppressing a subset of M1 markers. Further, MafB facilitates macrophage cholesterol efflux by directly up-regulating expression of Abca1 and Abcg1. Notably, we found that MafB expression is significantly elevated in response to a broad range of metabolic and immunological stimuli that promote macrophage M2 polarization, cholesterol efflux, or both, whereas it is strongly down-regulated in response to various pro-inflammatory pathogenic triggers. Further, MafB may mediate these signals in both LXR-dependent and independent way to promote macrophage cholesterol efflux. In contrast, MafB is directly targetable and down-regulated by pro-inflammatory and pro-atherogenic microRNAs, miR-155 and miR-33, which have been shown to be significantly up-regulated in atherosclerotic plaque^7^. These findings highlight physiological protective roles that MafB may play against atherosclerotic plaque progression and therapeutic potentials for targeting MafB to promote atherosclerotic regression.

## Results

### MafB promotes anti-inflammatory M2 polarization of macrophages

**Figure 1:**
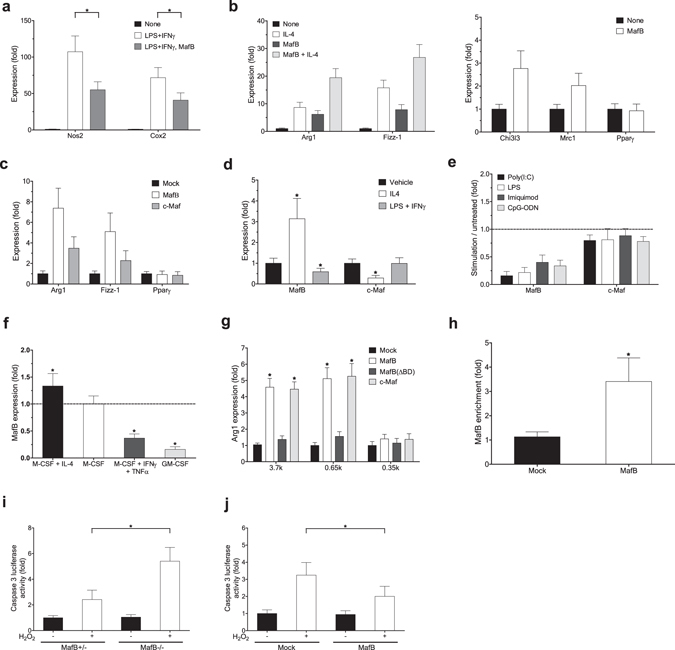
MafB promotes macrophage M2 polarization. (**a–f**) RT-PCR analysis. (**a,b**) Effect of ectopic MafB expression in mouse BMDM on M1/M2 polarization marker expression, measured at 24 h after stimulation. (**a**) M1 marker induction by LPS (10 ng/ml) and IFNγ (20 ng/ml). (**b**) M2 marker induction by IL-4 (40 ng/ml). (**c**) Effect of ectopic MafB expression in RAW264.7 cells on M2 markers. (**d**) Effect of mouse BMDM polarization on indicated gene expression. LPS plus IFNγ and IL-4 were treated for 24 h and 60 h, respectively. (**a–d**) Expression levels are presented relative to expression by vehicle- and/or mock-treated control. Sample number (n): n = 4. By two-tailed t-test, (**a,d**) *p < 0.05, (**b,c**) all except Pparγ: p < 0.05. (**e**) Effect of various pro-inflammatory stimulations in mouse BMDM. Agonists: poly(I:C) (5 µg/ml) (TLR3), LPS (100 ng/ml) (TLR4), imiquimod (2 µg/ml) (TLR7), and CpG-ODN (2.5 µg/ml) (TLR9). Expression levels are presented relative to vehicle-treated control samples. All MafB down-regulation: p < 0.05 (n = 4) by two-tailed paired sample t-test. (**f**) Effect of human monocyte-derived macrophages polarization on MafB expression. TNFα (20 ng/ml) plus IFNγ (M1) and IL-4 (M2) were treated for 24 h and 48 h, respectively. GM-CSF-derived macrophages (M1) were also compared. Expression levels are presented relative to M0 (M-CSF-derived) macrophages (*p < 0.05, n = 4). (**g**) Effects of MafB and c-Maf on activation of mouse *Arg1* promoter-derived reporters in 293ETN cells. 3.7k and 0.65k Arg1-Luc contain an AP-1 binding motif, whereas 0.35k does not. Luciferase activity was measured at 30 h after transfection. *p < 0.05, n = 8. (**h**) Effect of Flag-MafB expression on its recruitment to the *Arg1* promoter in RAW264.7 cells. Chromatin immunoprecipitation (ChIP) and RT-PCR. Promoter enrichments by anti-Flag antibody are presented relative to IgG-mediated enrichment. *p < 0.05, n = 4. (**i,j**) Effect of oxidative stress induced by H_2_O_2_ (200 μM) on cell apoptosis in MafB-deficient MEFs (**i**) and MafB-overexpressed RAW264.7 cells (**j**). Luciferase activity mediated by active caspase 3/7 was measured at 12 h post-stimulation. *p < 0.05, n = 8. (**a–j**) Data are represented as mean ± SD.

We previously found that MafB negatively regulates induction of Type I IFN and a subset of pro-inflammatory cytokines, and reduces cellular vulnerability to apoptosis induced by pro-inflammatory signaling^5^. In line with these, we found here that lentivirus-mediated transduction of MafB into mouse bone marrow derived macrophages (BMDM) considerably suppressed induction of a subset of marker genes of classically-activated, pro-inflammatory, M1 macrophages, including nitric oxide synthase 2 (Nos2) and cyclooxygenase 2 (Cox-2), upon treatment of M1-polarizing IFNγ and LPS (Fig. 1a). In addition, we found that MafB facilitates macrophage polarization into alternatively-activated, anti-inflammatory, M2 status. Ectopic MafB expression in untreated naïve (M0) BMDM strongly induced marker genes of M2 macrophages, including arginase-1 (Arg-1) and found in inflammatory zone 1 (Fizz-1), which was further potentiated by treatment of a M2 polarizing cytokine IL-4 (Fig. 1b). MafB also efficiently induced another M2 marker genes, Chitinase 3-like 3 (Chi3l3) and C-type mannose receptor 1 (Mrc1), but not a M2 promoting nuclear receptor, peroxisome proliferator activated receptor gamma (Pparγ). Similar results were obtained in RAW 264.7 mouse macrophage cell line (Fig. 1c). These findings suggest that MafB may broadly act to drive macrophages toward a less pro-inflammatory (M2) state.

In line with these functional roles of MafB, its expression was considerably down-regulated upon M1 polarization of mouse BMDM (Fig. 1d). Further, MafB expression in M0 BMDM was rapidly and strongly down-regulated in response to a variety of pro-inflammatory pathogenic stimuli (Fig. 1e), which is also consistent with our previous findings observed in various immune and non-immune tissues^5^. In contrast, MafB expression was significantly up-regulated upon IL-4 treatment to induce M2 polarization of mouse BMDM (Fig. 1d). Similar MafB expression patterns were observed in human primary macrophages that were derived from peripheral blood monocytes and then further polarized (Fig. 1f).

These results are in line with recent studies in human biological context reporting that MafB expression is considerably up-regulated in tumor-associated macrophages (TAM) that exhibit a M2-like functional profile^8^ and that MafB is required for efficient induction of M2 marker genes, including MRC1 and CD163, in human monocytes in response to IL-10 stimulation^9^.

Another member of the large Maf family of transcription factors, c-Maf, though weaker than MafB, could also induce the macrophage M2 marker genes in mouse macrophages (Fig. 1c). However, its expression pattern does not appear to be systematically associated with polarization and inflammatory status of macrophages (Fig. 1d and e).

We next investigated how MafB induces Arg-1 expression. Ectopic expression of MafB in 293ETN cells, in which expression of endogenous MafB is low^5^, strongly activated a transcriptional reporter driven by the promoter of mouse *Arg1* (3.7k upstream of the transcription start site (TSS)) (Fig. 1g and Supplementary Fig. S1). c-Maf could also equivalently induce Arg-1 expression. However, a DNA binding–defective mutant of MafB, MafB (ΔBD, N248S, *krENU* mutation)^5^, could not induce Arg-1 expression, suggesting that the DNA binding activity of MafB is a required element for its induction of Arg-1 expression. Notably, we found that MafB could similarly activate a shorter *Arg1* promoter (0.65k upstream of TSS) that contains only an AP-1 binding motif (TGACTCA at −375), but not other established binding motifs, including those of Irf8, Stat6, Klf4 and C/ebp^10^ (Supplementary Fig. S1). However, MafB failed to activate a further truncated *Arg1* promoter (0.35k upstream of TSS) that lacks the AP-1 motif (Supplementary Fig. S1). These, combined with our previous findings that MafB can effectively activate an AP-1 binding motif ^5^, suggest that the AP-1 motif is responsible for MafB-mediated activation of the *Arg1* promoter. Further supporting the notion, ectopically expressed MafB in RAW 264.7 cells was effectively recruited to the AP-1 motif (Fig. 1h).

In addition to protective roles of MafB against inflammation-induced apoptosis^5^, MafB appears to protect against cell apoptosis induced by elevated oxidative stress that has been highly implicated in the progression of atherosclerosis^11^. We assessed potential influences of MafB on apoptosis induced by hydrogen peroxide (H_2_O_2_) by measuring caspase-3 activity in mouse embryonic fibroblasts (MEFs) generated from homozygous Mafb-null (*Mafb* ^−/−^) and heterozygous (*Mafb* ^+/−^) mice^5^. Upon H_2_O_2_ treatment, caspase-3 activity was induced significantly higher in homozygous MEFs than in heterozygous MEFs (Fig. 1i). In line with this finding, ectopic MafB expression in RAW 264.7 cells significantly reduced H_2_O_2_-mediated induction of caspase-3 activity (Fig. 1j).

### MafB mediates IL-4/STAT6-dependent signaling

**Figure 2:**
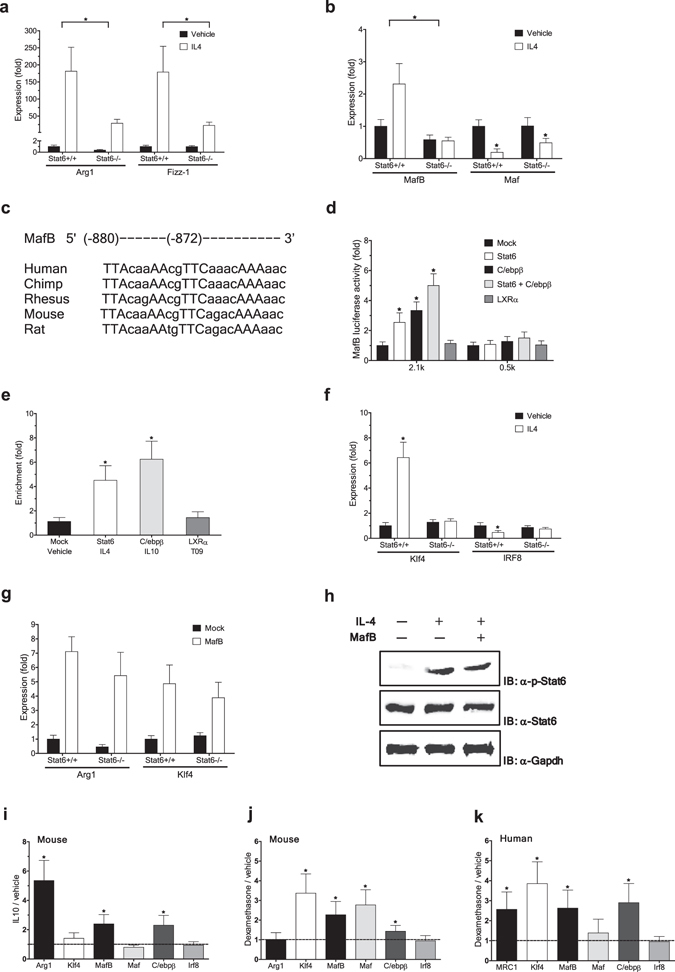
MafB mediates IL-4/STAT6-dependent signaling. (**a,b,f,g**) mouse BMDM from *Stat6* KO mice and their wild-type littermates were compared. (**a,b,f**) Effect of IL-4 treatment (60 h) on indicated gene expression (RT-PCR). Expression levels are presented relative to expression by vehicle treated wild-type control. *p < 0.05, n = 4. (**c**) Putative C/ebpβ and Stat6 binding sites on MafB promoters. (**d**) Luciferase activity of 293ETN cells transfected with human *MAFB* promoter-derived reporters, with or without ectopic expression of indicated genes. 2.1k contains putative binding sites for Stat6 and C/ebpβ, whereas 0.5k does not. Activity was measured at 36 h after transfection. *p < 0.05, n = 8. (**e**) Effect of ectopic expression of indicated genes (HA tagged) on their recruitment to the *MAFB* promoter in 293ETN cells (ChIP). *p < 0.05, n = 4. (**g**) Effect of ectopic MafB expression on indicated gene expression (RT-PCR at 30 h after transduction), presented as in a. All MafB-mediated induction: p < 0.05 (n = 4). (**h**) Effect of MafB on Stat6 phosphorylation. Immunoblot (IB) analyses in Stat6-overexpressed 293ETN cells. Each panel has been run separately under the same experimental condition. Un-cropped blots are available upon request. (**i–k**) Effects of M2 polarizing stimuli on expression of genes involved in M2 polarization and cholesterol efflux (RT-PCR). (**i**) IL-10 (40 ng/ml for 8 h) in mouse BMDM. (**j,k**) dexamethasone (Dex) (100 nM for 12 h) in mouse BMDM (**j**) and human MDM (**k**). (**i–k**) Expression levels are presented relative to expression by vehicle-treated control. *p < 0.05 (n = 4) by paired sample t-test. All: Data are represented as mean ± SD.

It has been well established that IL-4-induced polarization of macrophages to the M2 state depends critically on the signal transducer and activator of transcription 6 (Stat6) signaling pathway^12, 13^. Consistently, expression of M2 macrophage markers, Arg-1 and Fizz-1, was significantly reduced in BMDM derived from Stat6-deficient mice compared to wile-type BMDM: both basal and IL-4-induced levels (Fig. 2a). Notably, we found that expression of MafB, but not c-Maf, varies in parallel with the M2 markers (Fig. 2b), raising the possibility that MafB may be a downstream target of Stat6 that facilitates the Stat6-dependent polarization of macrophages into M2 status.

Further supporting the notion, we identified a putative Stat6 binding site (TTCaaacAAA at −872) on the human *MAFB* promoter, which is also conserved in mouse (TTCagacAAA at −921) (Fig. 2c and Supplementary Fig. S2). Further, ectopic expression of Stat6 in 293ETN cells, in which expression of endogenous Stat6 is low, could effectively activate a transcriptional reporter driven by the human *MAFB* promoter (2.1k upstream of TSS), whereas Stat6 failed to activate a shorter *MAFB* promoter (0.5k upstream of TSS) that does not contain the putative Stat6 binding site (Fig. 2d and Supplementary Fig. S2). Additionally, ectopic Stat6 expression, further upon IL-4 treatment, significantly increased Stat6 recruitment to the Stat6 motif (Fig. 2e). These findings suggest that MafB is a direct transcriptional target of Stat6.

The transcription factor Klf4 was found to cooperate with Stat6 to facilitate the macrophage M2 polarization^14^. Further, Klf4 was recently identified as a direct MafB transcriptional target in primary human epidermal progenitors^15^. In line with these, Klf4 expression was significantly induced upon IL-4 treatment in wild-type BMDM, but this induction was severely impaired in Stat6-deficient BMDM (Fig. 2f). However, expression of IRF8, another candidate positive regulator of Klf4 expression and M2 polarization^16^, was not induced, but rather decreased, upon IL-4 treatment in BMDM of both genotypes (Fig. 2f). Notably, ectopic MafB expression in Stat6-deficient BMDM effectively induced expression of M2 marker genes and Klf4 (Fig. 2g). These findings raise the possibility that MafB may mediate, at least in part, IL-4/Stat6-dependent signaling to induce expression of genes that promote the M2 polarization of macrophages. In addition, we found that MafB did not significantly affect IL4-induced activation (phosphorylation) of Stat6 (Fig. 2h). These findings support the view that MafB functions at a step downstream of the Stat6 phosphorylation to mediate IL-4/Stat6 signaling.

MafB appears to be broadly involved in various M2 type polarizations of macrophages. In addition to IL-4, MafB expression is strongly induced in response to other M2 polarizing stimuli. IL-10 is a potent anti-inflammatory cytokine that signals primarily through Stat3 activation^17^, which subsequently induces expression of multiple downstream transcriptional targets of Stat3, including CCAAT/enhancer-binding protein (C/ebp). IL-10 is also known to play important roles in the M2 polarization, and acts to induce Arg-1 expression^18^. Notably, upon IL-10 treatment, expression of MafB, as well as C/ebpβ and Arg-1, in mouse BMDM was significantly elevated (Fig. 2i). Bioinformatics analysis of the human *MAFB* promoter revealed a putative C/ebp binding site (TTAcaaAA at −880) in the very vicinity of the Stat6 motif (at −872) (Fig. 2c and Supplementary Fig. S2), which is also conserved in mouse (TTAcaaAA at −927). It is possible that this C/ebp-Stat6 composite DNA response element (TTAcaaAAcgTTCaaacAAA) plays a similar role to Stat6-C/ebp composite motifs found in many IL-4-responsive genes, including the mouse *Arg1* promoter (TTCttatGAAcaggctgtaTTAgccAAc)^19^. Notably, ectopic expression of C/ebpβ in 293ETN cells, in which expression of endogenous C/ebpβ is low, could effectively activate the human *MAFB* promoter containing the C/ebp motif (2.1k), but not a shorter *MAFB* promoter lacking this motif (0.5k) (Fig. 2d and Supplementary Fig. S2). Further, C/ebpβ acted in synergy with Stat6 to promote MafB expression. Additionally, ectopic C/ebpβ expression, further upon IL-10 treatment, significantly increased C/ebpβ recruitment to this motif (Fig. 2e). These findings suggest that MafB is a direct transcriptional target of C/ebpβ, as well as Stat6.

Glucocorticoids are also well established inducers of the M2 polarization. Treatment of mouse BMDM with a synthetic glucocorticoid dexamethasone (Dex) effectively induced expression of MafB and other reported mediators of the M2 polarization^12, 13^, although we could not detect any induction of Arg-1 expression (Fig. 2j). However, in human monocyte-derived macrophages (MDM), expression of a human M2 marker, the mannose receptor MRC1 (or CD206), as well as MafB, was effectively induced upon dexamethasone treatment (Fig. 2k). Expression of Stat6 did not exhibit any significant change in response to all the aforementioned treatments.

### MafB facilitates cholesterol efflux by upregulating ATP-binding cassette transporters

**Figure 3:**
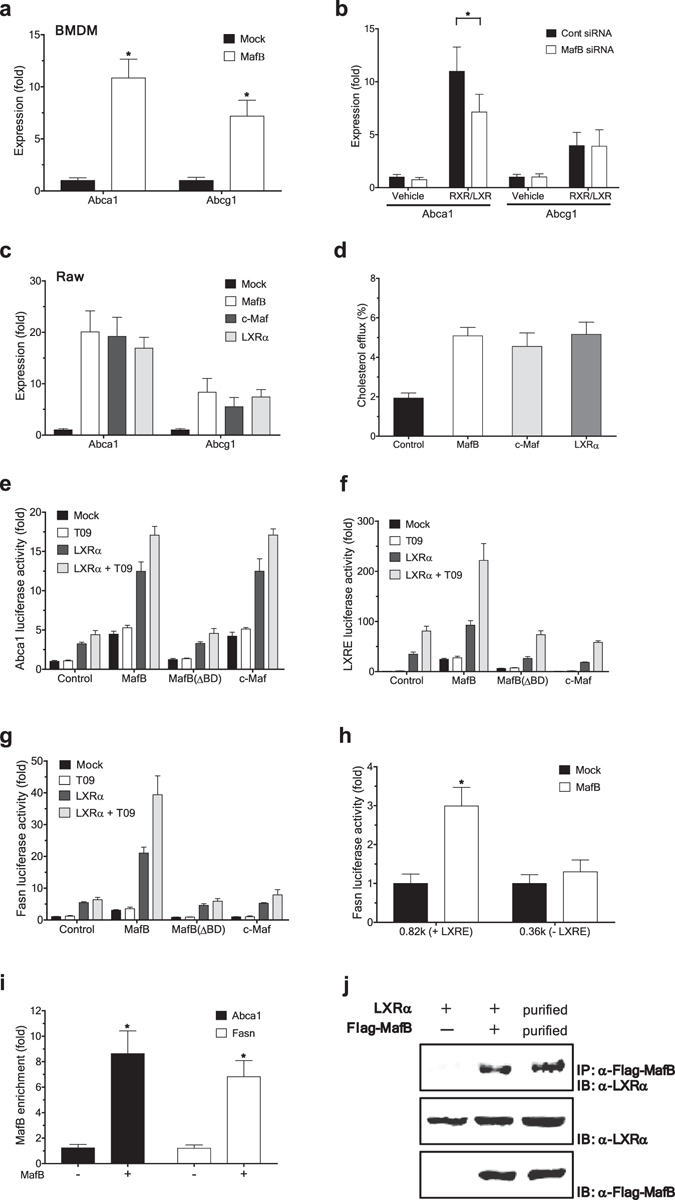
MafB facilitates cholesterol efflux by upregulating ATP-binding cassette transporters. (**a,b**) Effect of ectopic expression (**a**) or siRNA-mediated knockdown (**b**) of MafB on expression of Abca1 and Abcg1 in mouse BMDM (RT-PCR assay). Expression of ABC transporters was further induced using RXR and LXR agonists, 9cRA (0.5 µM) and GW3965 (1 μM), respectively. Expression levels are presented relative to expression by mock- and/or vehicle-treated control. *p < 0.05 (n = 4). (**c**) Effect of ectopic expression of an indicated gene on ABC transporter expression in RAW 264.7 cells (RT-PCR). Data are presented as in a. All: p < 0.05 (n = 4). (**d**) Effect of indicated gene overexpression on cholesterol efflux in J774A.1 cells. All: p < 0.05 (n = 6). (**e–g**) Effects of MafB, MafB (ΔBD) and c-Maf on activation of *ABCA1* and *FASN* promoters in 293ETN cells. Luciferase activity of cells transfected with a reporter of human *ABCA1* (**e**), LXRE (**f**) or *FASN* (**g**), with or without priming with LXRα co-expression. At 24 h after transfection, a LXRα agonist T0901317 (T09) (1 µM) was treated for 12 h. Activities are presented relative to expression by vehicle treated, mock-transfected control. n = 8. (**h**) Effect of MafB ectopic expression on activities of truncated *FASN* promoters (luciferase assay). n = 8. (**i**) Effect of Flag-MafB ectopic expression on its recruitment to the *ABCA1* and *FASN* promoters in 293ETN cells (ChIP). *p < 0.05, n = 4. (**j**) Interaction of Flag-MafB with LXRα (Immunoprecipitation (IP) analyses) in 293ETN cells. Interaction between immunopurified Flag-MafB and Myc/DDK-LXRα was also shown (third column). Each panel has been run separately under the same experimental condition. All: Data are represented as mean ± SD.

In addition to the roles of MafB to facilitate the M2 polarization of macrophages, MafB appears to have functional roles to promote cholesterol efflux in macrophages. We found that ectopic expression of MafB in mouse BMDM strongly induced expression of Abca1 and Abcg1, key mediators of cholesterol efflux (Fig. 3a). Consistently, knock-down of MafB considerably impaired Abca1 induction mediated by RXR and LXR agonists, 9cRA and GW3965, respectively (Fig. 3b). Similar results were obtained in RAW 264.7 cells (Fig. 3c). Notably, another large Maf transcription factor, c-Maf, could also induce expression of Abca1 and Abcg1, to levels comparable to those induced by MafB or LXRα (Fig. 3c). Accordingly, ectopic expression of MafB or c-Maf significantly enhanced cholesterol efflux in mouse macrophage J774A.1 cells (Fig. 3d), and similarly in RAW 264.7 cells (not shown).

We next explored how MafB regulates Abca1 expression. Ectopic expression of MafB in 293ETN cells strongly activated a transcriptional reporter driven by the human *ABCA1* promoter, to a level comparable to LXRα-induced ABCA1 activation (Fig. 3e). Additionally, MafB acted in synergy with LXRα to promote ABCA1 expression, which was further potentiated by treatment with a LXR agonist, T0901317 (T09). Notably, The MafB DNA binding–defective mutant could neither induce ABCA1 expression nor synergize with LXRα, suggesting that the DNA binding activity of MafB is a required element for its induction of ABCA1 expression. However, we could not detect any conventional MafB binding motif ^20^ in the vicinity of the proximal LXR-response element (LXRE), TGACCGatagTAACCT, in the human *ABCA1* promoter. Further, MafB could similarly activate a LXR specific reporter that contains only a multimerized LXR binding motif (TGACCAgcagTAACCT) (Fig. 3f). These findings suggest that MafB may be able to recognize a LXR binding motif, which bears sequence similarity to an established MafB binding motif (TGCTGACT)^15^, individually or in cooperation with LXR, to regulate expression of LXR transcriptional targets. Further supporting the notion, MafB could similarly activate the promoter of human fatty acid synthase (*FASN*) (0.82k upstream of TSS) (Fig. 3g and Supplementary Fig. S3), which also contains an established LXR binding motif (TGACCGgcagTAACCC). However, MafB failed to activate a shorter *FASN* promoter (0.36k upstream of TSS) that does not contain the LXRE (Fig. 3h and Supplementary Fig. S3). In line with these findings, ectopic expression of MafB in 293ETN cells strongly induced MafB recruitment to the LXR binding motifs (Fig. 3i). Further, MafB physically interacted with LXRα in these cells (Fig. 3j). This interaction appears to be direct since immunopurified MafB is also strongly associated with immunopurified LXRα (Fig. 3j).

We also found that c-Maf could similarly activate the *ABCA1* promoter (Fig. 3e). However, its synergistic interaction with LXRα could be observed only in the *ABCA1* promoter, but not in other LXRE-containing promoters (Fig. 3f and g), suggesting that roles of c-Maf in LXR-dependent transcriptional activation are rather limited.

### Cholesterol efflux-promoting signals upregulate MafB expression

**Figure 4:**
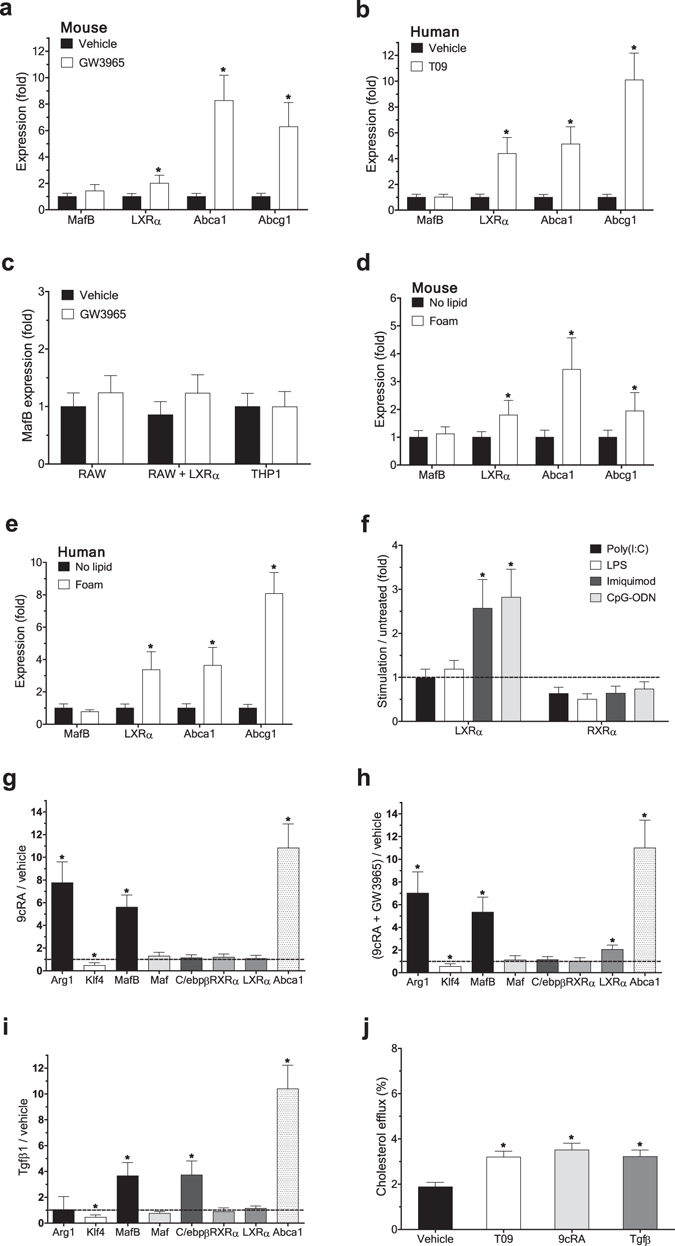
Cholesterol efflux-promoting signals upregulate MafB expression. (**a–c**) Effect of treatment of a LXR agonist, GW3965 (1 μM) or T09 (1 µM), for 18 h on expression of indicated genes in mouse BMDM (**a**), human MDM (**b**), and RAW 264.7 or THP1 cells (**c**) (RT-PCR). Expression levels are presented relative to expression by vehicle-treated control in each cell type. *p < 0.05, n = 4. (**d,e**) Effect of oxidized low-density lipoprotein (oxLDL) loading (for 24 h) on expression of indicated genes in mouse BMDM (**d**) and human MDM (**e**). Expression levels are presented relative to expression by no lipid loaded control. *p < 0.05, n = 4. (**f**) Effect of various pro-inflammatory stimuli in mouse BMDM on indicated gene expression. Expression levels are presented relative to expression by vehicle-treated control. *p < 0.05 (n = 4) by two-tailed paired sample t-test. (**g–i**) Effect of treatment of a RXR agonist, 9cRA (0.5 µM) (**g**), RXR and LXR agonists, 9cRA and GW3965 (**h**), or TGFβ (1 ng/ml) (**i**) for 18 h in mouse BMDM on indicated gene expression. Data are presented as in f. *p < 0.05, n = 4. (**j**) Effects of T09, 9cRA and TGFβ on cholesterol efflux in J774A.1 cells. *p < 0.05, n = 4. All: Data are represented as mean ± SD.

While this work was being completed, a potential role of LXR on MafB expression was reported^21^. This study also reported a putative LXR binding motif (CGTTCAaacaAAAACA) within the human *MAFB* promoter, which indeed overlaps with the Stat6 binding motif that we identified in the current work. Unlike this report, however, we could not detect any significant increase in MafB expression upon treatment with a LXR agonist, GW3965 or T09, in both mouse and human primary macrophages, while the same treatment significantly increased expression of LXRα, Abca1 and Abcg1 (Fig. 4a and b). Similar results were obtained in both mouse and human macrophage cell lines (Fig. 4c). Further, ectopic expression of LXRα in RAW264.7 (Fig. 4c) and 293ETN cells (Fig. 2d) could induce neither MafB expression nor recruitment of LXRα to the putative LXRE (Fig. 2e). Further supporting the notion, in both mouse and human primary macrophage foam cells loaded with oxidized low-density lipoprotein (oxLDL), an established LXR agonist, despite of significant increase in gene expression of LXRα, Abca1 and Abcg1, MafB expression remained uninduced (Fig. 4d and e). Additionally, LXR expression in mouse BMDM either remained unchanged or marginally increased in response to various pathogenic pro-inflammatory stimuli (Fig. 4f), while MafB expression was strongly down-regulated upon the same treatments (Fig. 1e).

It was previously reported that activation of retinoid-X receptor (RXR), a heterodimerization partner for various nuclear receptors including LXRα, induces expression of Abca1 and Abcg1, and facilitates cholesterol efflux in macrophages^22^. Further, RXR has been shown to play various anti-inflammatory roles in macrophages^23^. Consistently, treatment of mouse BMDM with a RXR agonist, 9cRA, significantly increased expression of Abca1 and Abcg1, as well as Arg-1 (Fig. 4g). Notably, the same treatments greatly increased MafB expression, whereas expression of LXRα, and also LXRβ (not shown), remained unchanged. Similar results were obtained when a LXR agonist GW3965 was co-treated with a RXR agonist (Fig. 4h). These findings suggest that MafB expression can be induced by both RXR homodimer and LXR/RXR heterodimer, but depends more critically on activity of RXR than LXR. These are indeed in line with a very recent report^24^ that RXR homodimer can positively regulate MafB expression in mouse osteoclast progenitors by directly binding to a RXR binding motif (DR-1), GAGTGTaAAGAGT at −367, in the *Mafb* promoter. However, this motif is not conserved in human, and we were unable to locate a functional RXR motif in the proximal region of the human *MAFB* promoter.

In addition, treatment of TGF-β in mouse BMDM, which was previously shown to induce Abca1 expression and cholesterol efflux in macrophage-derived foam cells^25^, significantly increased MafB expression, as well as Abca1 and Abcg1 expression, whereas LXRα expression remained unchanged (Fig. 4i). These findings suggest that LXR may not be a primary factor responsible for enhanced cholesterol efflux mediated by TGF-β, as well as RXR. Smad transcription factors are well established downstream effectors of TGF-β treatment. The mouse *Mafb* promoter contains an evolutionary conserved Smad binding motif (CCAGACA at + 235) in the 5′ untranslated region (UTR) (Supplementary Figs S2 and S4). However, this promoter also contains multiple un-conserved Smad motifs, and their exact roles remain to be clarified. Notably, the TGF-β treatment strongly induced C/ebpβ expression (Fig. 4i), adding an additional layer of stimulatory effects on MafB expression. We also confirmed that all the aforementioned treatments could efficiently induce cholesterol efflux in mouse macrophages (Fig. 4j).

Collectively, these findings support the view that MafB may mediate beneficial effects of a broad spectrum of metabolic and immunological signals to promote cholesterol efflux.

### Global view of regulation of MafB expression and its therapeutic implications

**Figure 5:**
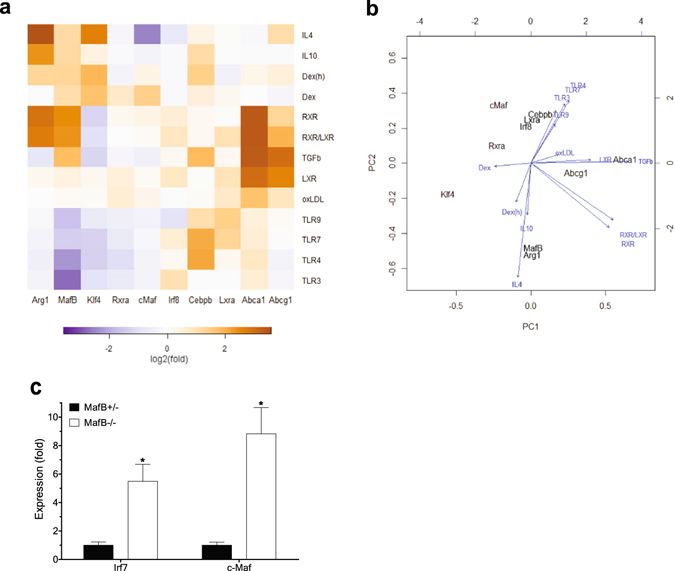
Global view of regulation of MafB expression and its therapeutic implications. (**a,b**) Integrative analyses of gene expression (RT-PCR, n = 4). Agonist used for each receptor is as follows. LXR: GW3965, RXR: 9cRA, TLR3: poly(I:C), TLR4: LPS, TLR7: imiquimod, and TLR9: CpG-ODN. Unless otherwise specified, results obtained in mouse BMDM were shown. Dex(h) indicates dexamethasone-treated human MDM, in which MRC1 expression was measured instead of Arg1. (**a**) Heatmap analysis. Row: different stimuli, Column: different genes. Log_2_ transformation of median fold changes (stimulation versus vehicle-treated) of expression levels of indicated genes were plotted. (**b**) Principal component analysis (PCA) and stimulation contribution plot. Principal component one (PC1) and two (PC2) primarily capture cholesterol efflux and M1/M2 polarization, respectively. Black: each gene in the PC space. Blue: contribution of each stimulus to the PCs. (**c**) Effect of MafB deficiency on c-Maf expression (RT-PCR). *Mafb* ^+/−^ and *Mafb* ^−/−^ MEFs were compared (*p < 0.05). Increase IRF7 was shown as a positive control.

To build a comprehensive view of the effects of various metabolic and immunological stimuli on macrophage polarization and cholesterol efflux, we examined and integrated expression profiles of all the aforementioned key regulators underlying the processes (Fig. 5a). One of the major observations is that although phenotypic shift of macrophages into an anti-inflammatory M2 state has largely been considered to have beneficial anti-atherogenic effects^2^, M2 polarization does not necessarily directly up-regulate genes that positively regulate cholesterol efflux (as shown with treatment of IL4, IL10 and Dex), and vice versa (as shown with treatment of TGFβ, LXR agonists and oxLDL), suggesting that M2 polarization and cholesterol efflux are two separate events. In addition, MafB expression was significantly induced in response to most of the stimuli that promote macrophage M2 polarization, cholesterol efflux, or both (a RXR agonist), with the exception of LXR agonists, whereas it was strongly down-regulated in response to various pro-inflammatory triggers that activate toll-like receptors (TLRs). Although MafB expression may not be directly regulated by LXR, MafB can act in synergy with LXR to potentiate LXR-dependent transcriptional induction of genes that promote cholesterol efflux (Fig. 3e). Klf4 expression varies in parallel with MafB expression in response to M2 polarizing and pro-inflammatory, but not cholesterol efflux-promoting, stimuli. C/ebpβ expression could be induced by most of both M1 and M2 polarizing stimuli. For the other genes examined, as well as Stat6 (not shown), no apparent systematic variation could be detected. These findings raise the intriguing possibility that MafB may be a key factor that broadly mediates anti-inflammatory, M2-polarizing, and cholesterol efflux-promoting effects in macrophages.

Principal component analysis (PCA) and stimulation contribution plot provides several novel insights (Fig. 5b): First, M2-polarizing (downward arrow, to the Arg1 direction) and cholesterol efflux-promoting (rightwards arrow, to the Abca1/Abcg1 direction) stimuli exert their effects in orthogonal directions, confirming further that these two processes represent two separate events. Second, a RXR agonist (down- and rightwards diagonal arrow) positively affects both M2 polarization (down side) and cholesterol efflux (right side). Third, pro-inflammatory stimuli (upward arrow) negatively regulate MafB expression (down side), whereas they positively regulate expression of C/ebpβ and LXRα. Fourth, both M2-polarizing stimuli (downward) and a RXR agonist (downward diagonal) were confirmed to positively regulate MafB expression. Sixth, expression pattern of MafB more closely resembles those of Arg1 (shorter downward direction distance between the two genes) and Abca1 (shorter rightward direction distance) than the other genes do. Seventh, c-Maf expression pattern is the least correlated with both the M2 polarization and cholesterol efflux (longer distance to Arg1 and Abca1, respectively). Collectively, these findings suggest that MafB may represent the primary form of the large Maf transcription factor that plays physiological roles in promoting both M2 polarization and cholesterol efflux in macrophages. Notably, we observed that c-Maf expression was significantly up-regulated in MafB-null MEFs (Fig. 5c), similar to what was previously observed in MafB-null macrophages^26^, raising the possibility that c-Maf may act to compensate, at least in part, for the elevated pro-inflammatory^5^ and pro-atherogenic risks caused by MafB deficiency.

It has been suggested that excess cholesterol accumulation in macrophages and accompanying intracellular pro-inflammatory responses may exert inhibitory effects on LXR activity, which in turn may impair cholesterol efflux from macrophage foam cells^27^. However, we found that in both human and mouse macrophage foam cells, expression of LXR targets Abca1 and Abcg1, as well as LXR itself, was not down-regulated, but rather significantly increased (Figs 4d,e and 5a), suggesting that LXR activity to induce cholesterol efflux was not significantly impaired. Further, LXR expression in macrophages did not decrease in response to various pro-inflammatory stimuli (Figs 4f and 5a). Instead, we found that MafB expression was strongly inhibited by elevated pro-inflammatory signaling (Figs 1e and 5a), raising the intriguing possibility that this reduced MafB activity may actually be a key factor responsible for inflammation-mediated deterioration in macrophage cholesterol efflux. Furthermore, the findings that MafB expression remained suppressed in macrophage foam cells, in which LXR activity may already have reached its upper limit, may provide exciting novel therapeutic opportunities to further improve cholesterol efflux via enhancing MafB-dependent signaling, which in turn may boost both LXR-dependent and independent anti-atherogenic signaling.

In line with this notion, analyses of publically available high-throughput datasets^28^ revealed that in a mouse model of atherosclerosis regression, expression of MafB, as well as Abca1, in plaque macrophages was substantially up-regulated during atherosclerosis regression (Supplementary Fig. S5), suggesting potential anti-atherogenic roles of MafB.

### Atherogenic miR-155 and miR-33 negatively regulate MafB

MicroRNAs are small, noncoding RNAs that directly bind to the 3′ UTR of target mRNAs to degrade them or inhibit their translation. It has been shown that miR-155 expression is significantly up-regulated in both human and mouse macrophages under pro-inflammatory M1-polarizing conditions or in response to various pro-inflammatory stimuli^7^. miR-155 plays roles in promoting macrophage pro-inflammatory responses by directly targeting and suppressing their negative regulators^7^, including BCL6, SOCS1 and SHIP1, which in turn might promote the development of atherosclerosis^29^. In addition, miR-155 was reported to suppress expression of C/ebpβ^30^, a positive regulator of expression of Arg-1, as well as MafB (Fig. 2d), driving macrophages further toward an elevated pro-inflammatory state.

**Figure 6:**
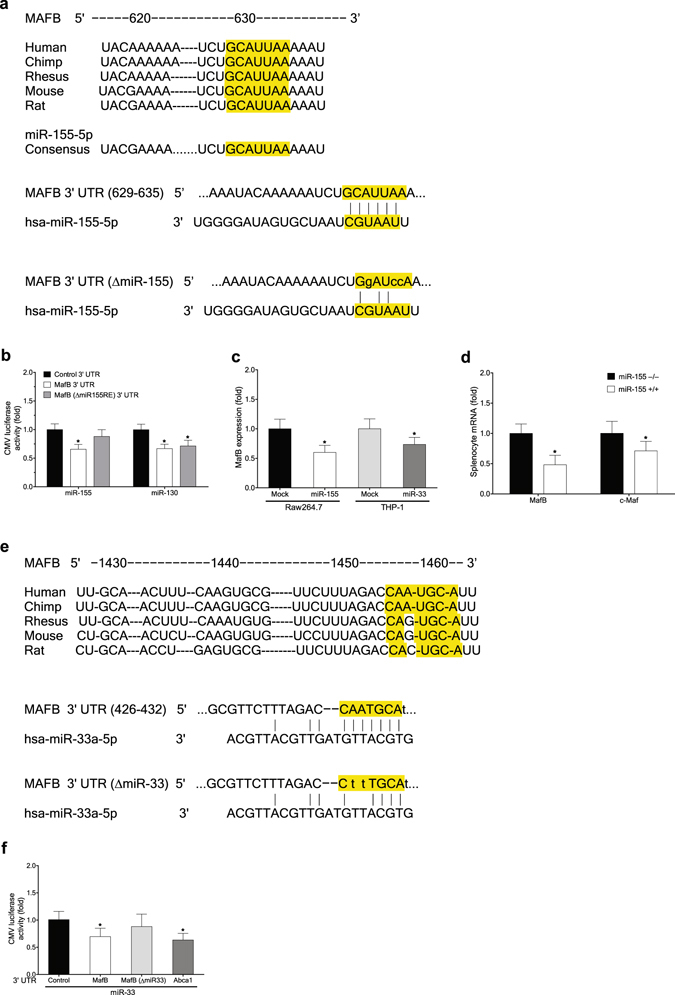
Atherogenic miR-155 and miR-33 negatively regulate MafB. (**a**) Sequence of MafB 3′ UTR targeted by miR-155 (TargetScan). (**b**) Effect of miR-155 ectopic expression in 293ETN cells on activities of a MafB 3′ UTR luciferase reporter and its mutant non-targetable by miR-155 (luciferase assay). Effect of miR-130 ectopic expression was compared as a positive control. *p < 0.05, n = 8. (**c**) Effects of ectopic expression of miR-155 (left) and miR-33 (right) on the endogenous level of *MafB* mRNA in RAW 264.7 (left) and THP-1 cells (right) (RT-PCR assay). *p < 0.05, n = 4. (**d**) Effect of miR-155 deficiency on the endogenous level of *MafB* mRNA (RT-PCR). Splenocytes isolated from miR-155-deficient mice were compared to those from wild-type mice. *p < 0.05, n = 4. (**e**) Sequence of MafB 3′ UTR targeted by miR-33 (TargetScan). (**f**) Effect of miR-33 ectopic expression in 293ETN cells on activities of MafB 3′ UTR luciferase reporters. Effect on an ABCA1 3′ UTR reporter was shown as a positive control. *p < 0.05, n = 8. All: Data are represented as mean ± SD.

To explore the possibility that miR-155 may also exert its pro-inflammatory and pro-atherogenic effects via MafB down-regulation, we examined whether MafB can be directly targeted by miR-155. Bioinformatics analysis of the 3′ UTR of MafB mRNA suggested that it contains a highly conserved candidate sequence targetable by miR-155 (Fig. 6a). Accordingly, by using a CMV promoter-driven reporter harboring the 3′ UTR of MafB mRNA, we found that MafB expression could be inhibited by co-expressed miR-155 (Fig. 6b), to an extent similar to inhibition mediated by miR-130, a previously reported MafB-targeting microRNA^31^. However, a non-targetable mutation of the candidate miR-155 target sequence blocked the inhibitory effect of miR-155. Further, ectopic expression of miR-155 in RAW 264.7 cells significantly lowered the endogenous level of MafB mRNA (Fig. 6c). In agreement with these findings, we could detect significantly elevated levels of expression of MafB, to a lesser extent c-Maf ^32^, a previously reported target of miR-155, in splenocytes isolated from miR-155-deficient mice compared to their wild-type littermates (Fig. 6d). These findings indicate that MafB mRNA is a direct target of miR-155. Further, down-regulation of MafB expression upon macrophage M1 polarization or in response to various pro-inflammatory stimuli may be attributed, at least in part, to elevated miR-155 expression under these conditions.

miR-33, an intronic microRNA located within the SREBF2 gene, has been shown to target and suppress Abca1 expression in human and mouse macrophages, and thereby attenuate reverse cholesterol transport and promote atherosclerotic development^33^. Notably, we found that this pro-atherogenic miR-33 could directly target the 3′ UTR of MafB mRNA in human (Fig. 6e), although candidate miR-33 target sequence was not fully conserved in mouse. Accordingly, ectopic miR-33 expression in 293ETN cells significantly reduced the activity of the MafB 3′ UTR reporter, to an extent equivalent to an ABCA1 3′ UTR reporter (Fig. 6f). However, a non-targetable mutation of the candidate miR-33 target sequence blocked the inhibitory effect of miR-33. Further, ectopic expression of miR-33 in THP-1 cells significantly reduced the endogenous MafB mRNA (Fig. 6c), indicating that human MafB mRNA is a direct target of miR-33. Notably, analyses of publically available high-throughput datasets revealed that in two separate studies of african green monkeys^34, 35^, *in vivo* administration of anti-miR-33 significantly increased hepatic expression of MafB (Supplementary Figs. S6 and S7), as well as a known miR-33 target, Abca1, despite A to G substitution in the miR-33 seed region of MafB 3′ UTR in these animals (Fig. 6e). However, the same treatments did not enhance expression of c-Maf and aforementioned potential positive regulators of MafB that do not contain candidate miR-33 target sequence in their 3′ UTRs. These findings suggest that the increase in MafB expression may be attributed largely to reduced extent of miR-33-mediated inhibition, further supporting the notion that MafB may also be a direct target of miR-33 in these animals.

Collectively, these findings support the view that MafB may be broadly targeted by pro-inflammatory and pro-atherogenic microRNAs, and the resulting deterioration of protective MafB activities in turn may increase host vulnerability to the development of atherosclerosis.

## Discussion

The biological functions of MafB and its gene expression and regulatory patterns uncovered in this work support the view that in addition to its roles in establishing the monocyte-macrophage lineage^6^, MafB plays pivotal roles in regulating macrophage polarization. MafB acts to enhance expression of anti-inflammatory M2 polarization marker genes in macrophages, while it suppresses expression of a subset of pro-inflammatory M1 marker genes, thereby driving macrophage phenotype into a less pro-inflammatory (M2) state. Further, MafB enhances expression of Abca1 and Abcg1, and thereby facilitates cholesterol efflux from macrophage foam cells. Notably, MafB can act in synergy with LXRα or in a LXR-independent manner to promote Abca1 expression. In contrast, MafB is directly targeted and down-regulated by pro-inflammatory and pro-atherogenic microRNAs, miR-155 and miR-33. These findings suggest that MafB may play physiological roles in protecting against the development of atherosclerosis.

Macrophages play pivotal roles in the progression and regression of atherosclerosis, and their inflammatory phenotypes have been suggested as key determinants of the course of the disease^2^. Accumulating evidence suggests that phenotypic shift of plaque macrophages, from a pro-inflammatory M1 to an anti-inflammatory M2 state, may be a key characteristic of atherosclerotic plaques undergoing regression. In the present work, using an integrative systems biology approach, we uncovered that M2 polarization does not necessarily have a direct promoting effect on cholesterol efflux (Fig. 5a and b), and vice versa, suggesting that these two processes represent two separate events. Notably, we found that MafB expression is significantly elevated in response to most of the stimuli that promote macrophage M2 polarization, cholesterol efflux, or both, with the exception of LXR agonists, whereas it was strongly down-regulated in response to various pro-inflammatory triggers. Further, despite the LXR-independent nature of MafB expression, MafB could synergize with LXR to potentiate LXR-dependent induction cholesterol efflux (Fig. 3e). These findings support the view that MafB may be broadly involved in intracellular anti-inflammatory, M2-polarizing and cholesterol efflux-promoting signaling pathways in macrophages, by which MafB may play protective roles against atherosclerotic progression.

While this work was being completed, two interesting studies on potential roles of MafB in the development of atherosclerosis have been reported^21, 36^. In early stages of atherosclerosis, enhanced apoptosis of cholesterol-laden macrophages may have protective roles against atherosclerotic lesion growth since these apoptotic cells can be efficiently cleared by the neighboring macrophages, via a process called efferocytosis^37^. In this stage, MafB was reported to play atherosclerosis promoting roles by up-regulating apoptosis inhibitor of macrophages (AIM) and thereby suppressing apoptosis of macrophage foam-cells^21^. In contrast, in advanced stages of atherosclerosis, macrophage apoptosis is greatly increased in necrotic atherosclerosis lesions, due to elevated cholesterol-induced cytotoxicity and endoplasmic reticulum stress in macrophages, while clearance of these apoptotic cells by efferocytosis is severely impaired. These uncleared apoptotic cells subsequently induce various pro-inflammatory responses in atherosclerotic lesions, promoting further the development and expansion of necrotic cores. In this stage, MafB was reported to play protective roles against deleterious atherosclerotic progression by suppressing macrophage apoptosis while enhancing atherosclerotic plaque stability^36^. Our findings support and extent these studies by showing that in addition to anti-inflammatory and anti-apoptotic roles of MafB, it can directly up-regulate expression of Abca1 and Abcg1 in macrophages by mediating a broad spectrum of anti-inflammatory and anti-atherogenic signals, and thereby promote cholesterol efflux, which in turn may provide protection against atherosclerotic progression.

The Maf transcription factors can be classified into two subfamilies, the large and small Maf families, in which the former contains a transcriptional activation domain. The large Maf family consists of MafB, c-Maf, MafA and NrL. Among these, MafB, and to a lesser extent c-Maf, is expressed highly in monocyte and macrophage lineages of the hematopoietic system^38^. Further, MafB and c-Maf have been reported to have some overlapping functional roles in macrophages, including positive regulation of F4/80 gene expression^21^. Our current work extents these studies by showing that both MafB and c-Maf can directly up-regulate expression of key mediators of cholesterol efflux, including Abca1 and Abcg1, as well as M2 polarization markers, in macrophages. Further, we showed that MafB can act in synergy with LXRα over a broad range of LXRE-containing promoters, whereas synergistic interaction between c-Maf and LXRα appears to be rather limited. Importantly, MafB and c-Maf exhibited marked differences in their gene expression and regulatory patterns in response to various inflammatory and atherogenic stimuli (Fig. 5a). While MafB expression profile is strongly correlated with inflammatory and atherogenic phenotypes of macrophages, no such correlation could be detected for c-Maf. Additionally, no significant correlation was apparent for other established regulators of macrophage polarization and cholesterol efflux. Notably, MafB, but not c-Maf, is directly targetable and negatively regulated by pro-atherogenic miR-33 (Fig. 6f). Further, we observed that MafB could be more effectively targeted by another pro-atherogenic microRNA, miR-155, than c-Maf (Fig. 6d). These findings suggest that MafB may be a more physiologically relevant mediator of anti-inflammatory and anti-atherogenic signals in macrophages. Notably, in the aforementioned study^21^, no significant difference in Abca1 and Abcg1 expression was observed between MafB-deficient and wild-type macrophages. This may be attributed, at least in part, to compensatory up-regulation of c-Maf expression in MafB-deficient cells, which we and others have observed in MafB-null MEFs (Fig. 5c)^5^ and macrophages^26^.

Collectively, we showed that MafB is a key intracellular mediator of a broad range of anti-inflammatory and anti-atherogenic signaling in macrophages. Further, our findings that MafB can promote macrophage cholesterol efflux in both LXR-dependent and independent manners, combined with the findings that MafB expression remains suppressed in oxidized cholesterol-laden or pro-inflammatory macrophages, may offer novel therapeutic opportunities to improve current treatment strategies for atherosclerosis via enhancing MafB-dependent signaling.

## Methods

### Animal studies

All experimental procedures were approved by the Massachusetts General Hospital Committee on Research Animal Care and conducted in accordance with the institutional guidelines and regulations. Homozygous Stat6 (B6.129S2(C)-Stat6tm1Gru/J) and miR-155 (B6.Cg-Mirn155tm1.1Rsky/J) null mice were purchased from The Jackson Laboratory. Unless otherwise specified, results for male mice were shown.

### Plasmids and mutagenesis

Human and mouse expression plasmids were from Open Biosystems or OriGene unless otherwise specified. Full-length or truncated open reading frames (ORF) from plasmids were subcloned into pCMV, Flag-pCMV or HA-pCMV^5^. Truncation and DNA binding-deficient mutants were generated by site-directed mutagenesis PCR. For microRNA targeting, the CMV promoter and 3′UTRs of indicated genes were subcloned into pGL4 (Promega). For MafB lentivirus, mouse MafB ORF was subcloned into CSGW vector. Transcriptional reporters were generated as summarized in Supplementary Table S1 ^5^.

### Cell culture, Transfection, Virus Infection and Luciferase assay

Detailed procedures are described in our previous work^5^. RAW 264.7 and THP-1 cells are transfected using TurboFect transfection reagents (Thermo Fisher Scientific) and the Nucleofector™ kit (Lonza), respectively, according to the manufacturer’s recommendations. For transfection of MafB siRNA (156036, Ambion) (50 nM), X-tremeGENE Transfection Reagents (Roche) was used.

### Human monocyte-derived macrophages and mouse bone marrow derived cells

Healthy human blood samples were obtained from MGH, and written informed consent was obtained from all blood donors. All experimental procedures were approved by the MGH Institutional Review Board and conducted in accordance with the institutional guidelines and regulations. Human peripheral blood mononuclear cells (PBMCs) were isolated from the buffy coat of blood samples using Lymphoprep™ (STEMCELL Technologies). Monocytes were enriched using CD14 positive magnetic selection (Miltenyi Biotec), and then cultured in complete RPMI 1640 medium containing 10% FBS, supplemented with 50 ng/ml of human M-CSF (300-25; Peprotech) for 7 days. The culture media were changed every two days. Mouse bone marrow was harvested from the femurs and tibias of 11-week old mice, and cultured for 7 days in RPMI-1640 containing 10% FBS, supplemented with 15 ng/ml of murine M-CSF (315-02; Peprotech) or murine GM-CSF (315-03; Peprotech) for bone marrow derived macrophages (BMDM) and bone marrow derived dendritic cells (BMDC), respectively. The culture media were changed every two days. On day 7, the cells were further treated with IFNγ (20 ng/ml), TNFα (20 ng/ml), LPS (10 ng/ml) and/or IL-4 (40 ng/ml). To induce foam cell formation, the cells were treated with human oxidized low density lipoprotein (oxLDL) (Kalen Biomedical) (50 μg/mL) for 24 h.

### Quantitative real-time PCR, Immunoblot and Chromatin immunoprecipitation

All cells and tissues were processed and analyzed as previously described^5^. For RT-PCR, total RNA was extracted using a TRIzol extraction method. The sequences of primers for RT-PCR and ChIP assays are listed in Supplementary Tables S2 and S3 ^5^. Flag-tagged MafB was purified with M2 Affinity Gel as previously described^5^, and Myc/DDK-tagged LXRα was obtained from OriGene. Immunoblot and ChIP analyses used the following antibodies: anti-β-actin (ab8226; Abcam), anti-GAPDH (sc-25778; Santa Cruz), anti-MafB (MAB3810; R&D Systems), anti-Stat6 (sc-621; Santa Cruz), anti-pStat6 (sc-11762; Santa Cruz), anti-LXRα (ab41902; abcam), anti-Flag (M2) or anti-HA Affinity Gel (Sigma), goat anti-rabbit IgG-HRP (sc-2054; Santa Cruz), IRDye 800CW–conjugated goat anti–mouse and anti–rabbit IgG (926-32210 and 926-32211, respectively; LI-COR), and IRDye 800–conjugated anti-Flag and anti-HA (600-432-383 and 600-432-384, respectively; Rockland). Immunocomplexes were visualized with an Odyssey Infrared Imaging System (LI-COR).

### Cholesterol efflux assay

RAW264.7, J774A.1 cells or mouse BMDM were seeded in 24-well plates (10^5^ cells/well) in complete DMEM medium containing 10% FBS. Where indicated, the cells were transfected with an indicated plasmid for 24 h, followed by PBS washing. The cells were then incubated in the complete medium supplemented further with [^3^H]-cholesterol (1 μCi/ml) for 24 h. The cells were washed with PBS and then incubated in DMEM medium supplemented with 0.2% fatty acid free-BSA for 2 h. The cells were rinsed with PBS again, and then incubated in the DMEM-0.2% BSA medium. To determine cholesterol efflux, ApoAI (10 μg/ml) was added to the medium for 24 h. Where indicated, the cells were co-treated with an indicated compound. Subsequently, the medium was collected and centrifuged to remove cell debris. The cells were washed with PBS, and then lysed in 0.1 N NaOH. Radio-activities of the medium and the cell lysate were measured by liquid scintillation counting. Cholesterol efflux was expressed as the percentage of radioactivity in the medium relative to the sum of those in the cell lysate and the medium.

### Caspase activity assay

Caspase 3/7 activity was measured with the Caspase-Glo 3/7 Assay kit (Promega) as previously described^5^.

### Statistics, integrative data analysis and microarray analysis

Unless otherwise specified, a two-tailed Student’s t-test was applied for the comparison of two independent data sets. A two-tailed paired sample t-test was applied for the comparison of data sets from stimulation versus vehicle treated samples; p values of 0.05 were considered statistically significant. Heatmap and principal component analysis (PCA) were performed using the R Project for Statistical Computing^39, 40^. Microarray datasets were processed using R, and expression profiles were summarized using the GC Robust Multi-array Average (GCRMA) method as implemented in Bioconductor (http://www.bioconductor.org)^5^.

## Electronic supplementary material

Supplementary Information
