# Supplementary material for: The transcription factor MafB promotes anti-inflammatory M2 polarization and cholesterol efflux in macrophages
Source: Sci Rep. 2017 Aug 8;7:7591. doi: 10.1038/s41598-017-07381-8 (PMC5548719; doi:10.1038/s41598-017-07381-8)
Supplement: Supplementary file 1 — Supplementary Information [file 41598_2017_7381_MOESM1_ESM.pdf]

Supplementary Information

The transcription factor MafB promotes anti-inflammatory M2 polarization and cholesterol efflux in macrophages

Hwijin Kim*

Center for Computational and Integrative Biology, Massachusetts General Hospital, Boston MA 02114, USA

Department of Genetics

Harvard Medical School, Boston MA 02115, USA

Phone: 617-643-3340. Fax: 617-643-3328. Email: hwijinkim@gmail.com

*Corresponding Author

Supplementary Figure Legends

Fig. S1. Organization of mouse Arg-1 promoter

Fig. S2. Organization of human MafB promoter

Fig. S3. Organization of human FASN promoter

Fig. S4. Putative Smad binding site on MafB promoters.

Fig. S5. Expression of the MAF family members and key mediators of M2 polarization and cholesterol efflux in plaque macrophages of the Reversa mouse model of atherosclerosis regression[1]. In this model, poly(I:C) injections induce recombination-mediated inactivation of Mttp in the liver, which in turn acts to restore normal lipid levels and induce atherosclerotic plaque regression in atherosclerosis mice.

Fig. S6. Effect of anti-miR-33 treatment on hepatic gene expression in african green monkeys[2].

Fig. S7. Effect of treatment of a miR-33a/b-targeting LNA-antimiR on hepatic gene expression in african green monkeys[3].

Fig. S1.

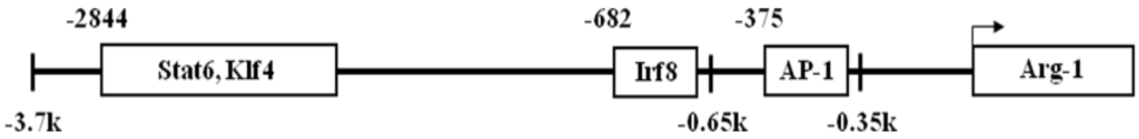

Fig. S2.

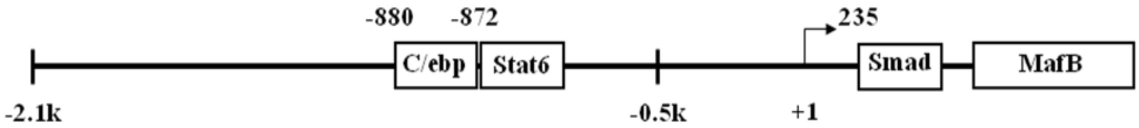

Fig. S3.

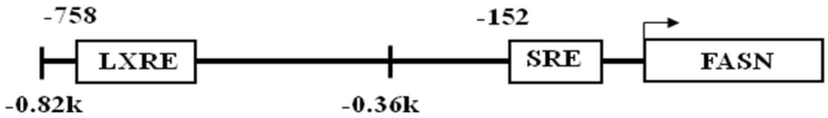

Fig. S4.

MafB 5' (+235)———————— 3’

Human tccCCAGACAaag
Chimp tccCCAGACAaag
Rhesus ——————————————
Mouse tccCCAGACAaag
Rat tccCCAGACAaag

Fig. S5.

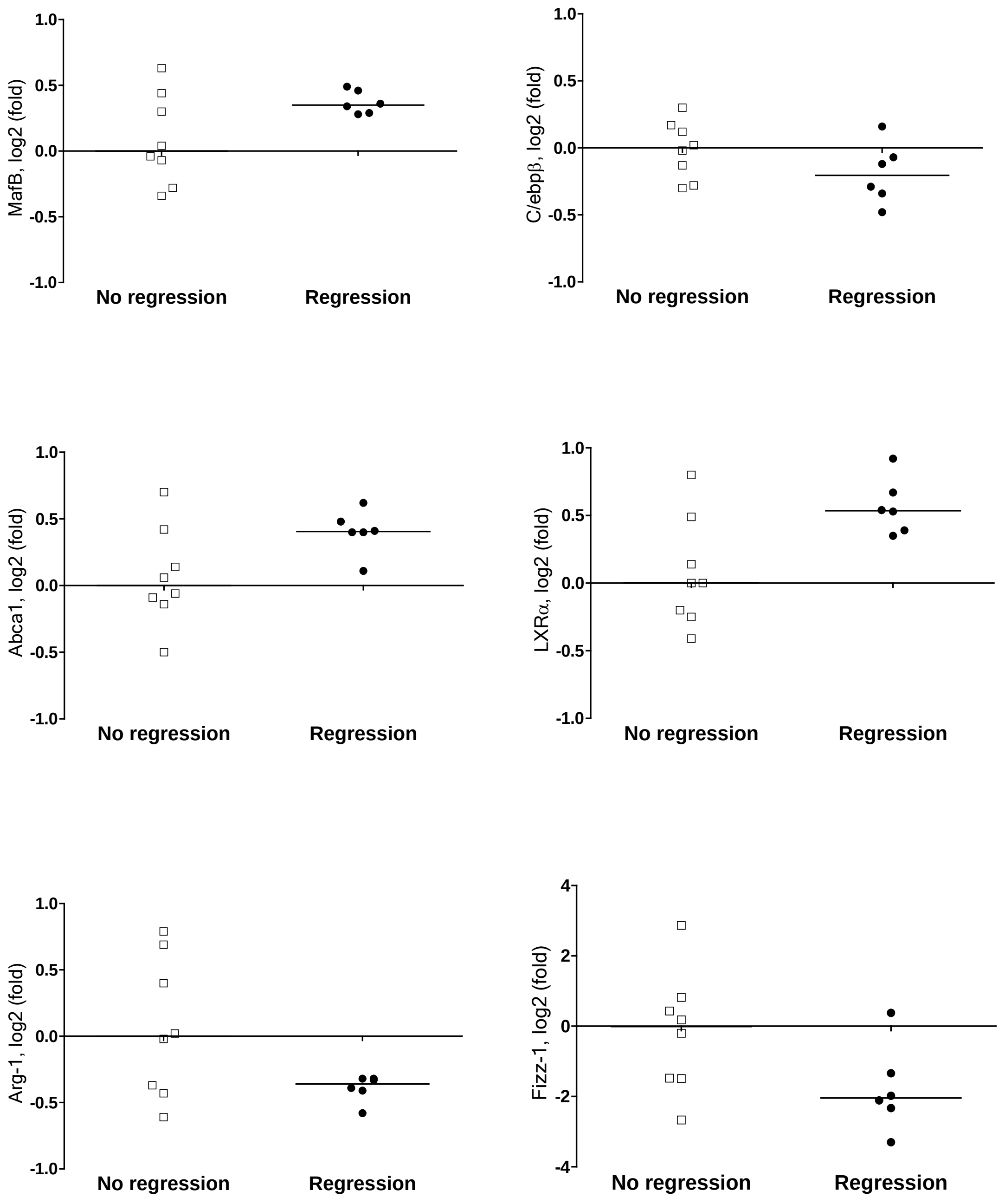
MafB, log2 (fold)
1.0
0.5
0.0
-0.5
-1.0
No regression
Regression
C/ebpβ, log2 (fold)
1.0
0.5
0.0
-0.5
-1.0
No regression
Regression
Abca1, log2 (fold)
1.0
0.5
0.0
-0.5
-1.0
No regression
Regression
LXRα, log2 (fold)
1.0
0.5
0.0
-0.5
-1.0
No regression
Regression
Arg-1, log2 (fold)
1.0
0.5
0.0
-0.5
-1.0
No regression
Regression
Fizz-1, log2 (fold)
4
2
0
-2
-4
No regression
Regression

Fig. S6.

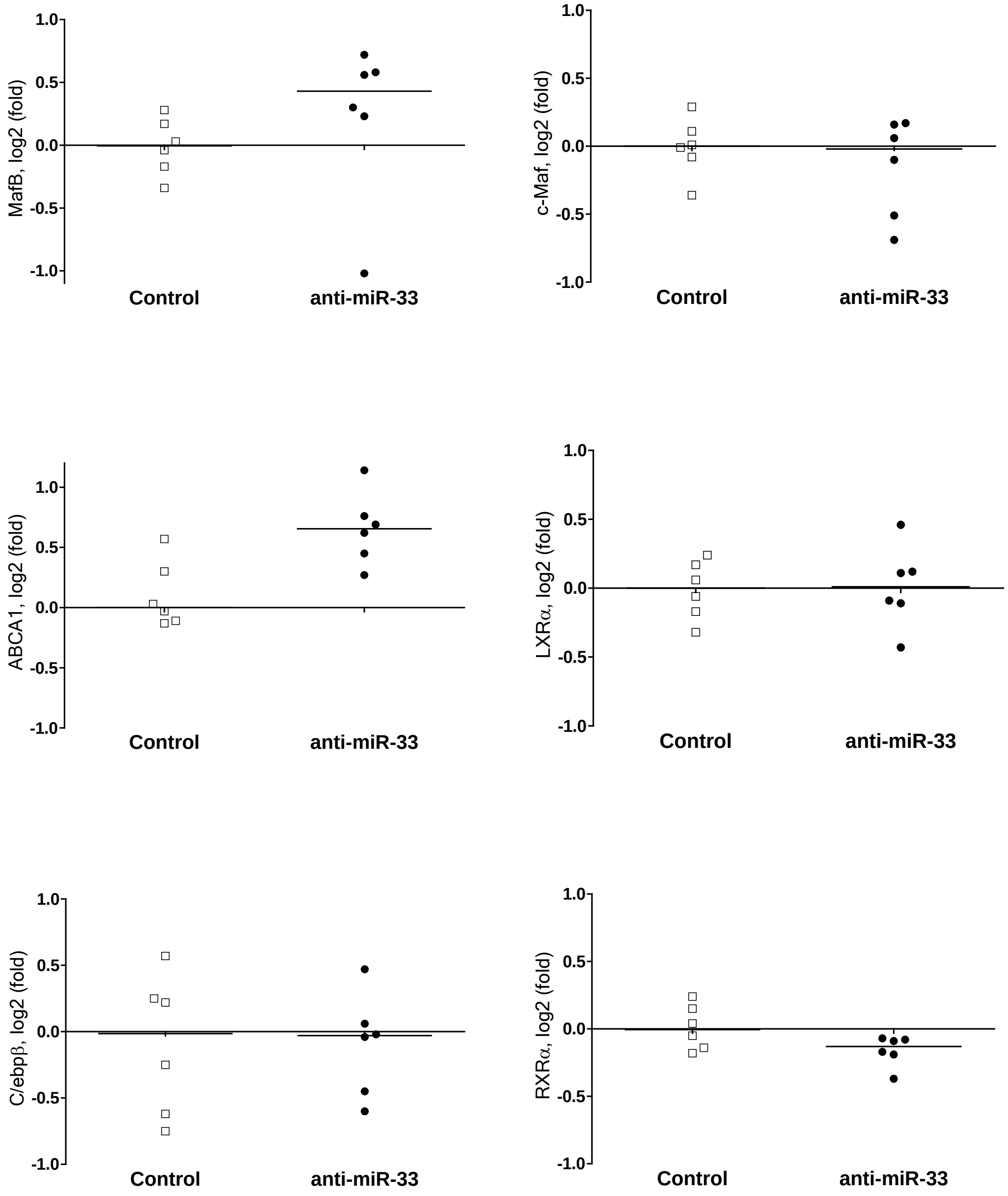
MafB, log2 (fold)
1.0
0.5
0.0
-0.5
-1.0
Control
anti-miR-33
c-Maf, log2 (fold)
1.0
0.5
0.0
-0.5
-1.0
Control
anti-miR-33
ABCA1, log2 (fold)
1.0
0.5
0.0
-0.5
-1.0
Control
anti-miR-33
LXRα, log2 (fold)
1.0
0.5
0.0
-0.5
-1.0
Control
anti-miR-33
C/ebpβ, log2 (fold)
1.0
0.5
0.0
-0.5
-1.0
Control
anti-miR-33
RXRα, log2 (fold)
1.0
0.5
0.0
-0.5
-1.0
Control
anti-miR-33

Fig. S7.

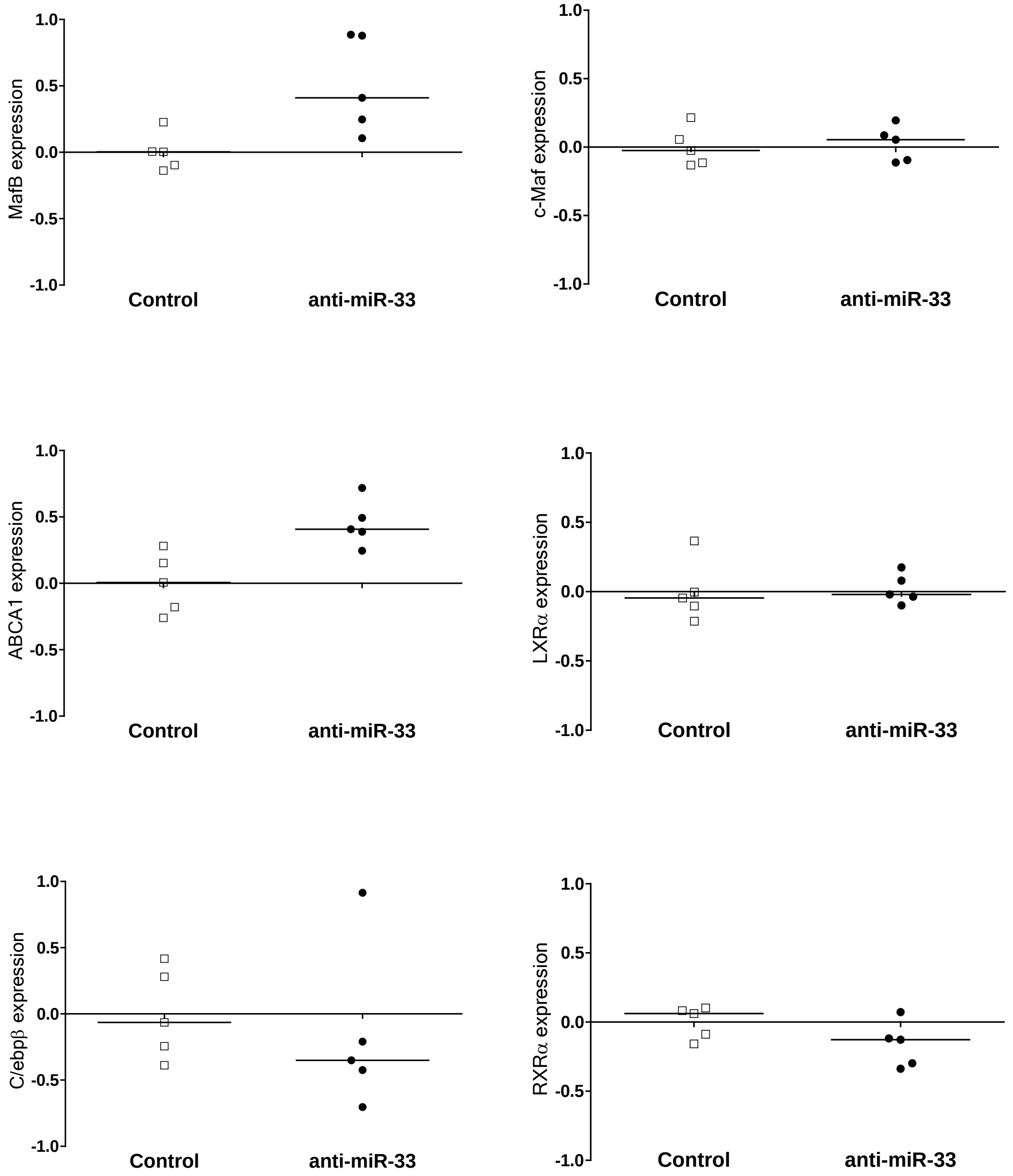
MafB expression
1.0
0.5
0.0
-0.5
-1.0
Control
anti-miR-33
c-Maf expression
1.0
0.5
0.0
-0.5
-1.0
Control
anti-miR-33
ABCA1 expression
1.0
0.5
0.0
-0.5
-1.0
Control
anti-miR-33
LXRα expression
1.0
0.5
0.0
-0.5
-1.0
Control
anti-miR-33
C/ebpβ expression
1.0
0.5
0.0
-0.5
-1.0
Control
anti-miR-33
RXRα expression
1.0
0.5
0.0
-0.5
-1.0
Control
anti-miR-33

Reporter	Transcription factor response element
MafB-Luc	Human MafB promoter (–2077 to +391)
ABCA1-Luc	Human ABCA1 promoter (–808 to +149)
LXRE-Luc	4x LXR responsive element, plus sTATA
FASN-Luc	Human FASN promoter (–823 to +56)
Arg1-Luc	Mouse Arg1 promoter (–3698 to +74)

Supplementary Table S1. Transcriptional luciferase reporters. An indicated response element was inserted into the pGL4 vector (Promega).

Mouse Gene	Direction	Sequence (5’ → 3’)
Gapdh	f	catcactgccacccagaagactg
	b	atgccagtgagcttcccgttcag
mouse/human MafB	f	gacgcagctcattcagcag
	b	ccggagttggcgagtttct
c-Maf	f	agcagttggtgaccatgtcg
	b	tggagatctcctgcttgagg
Arg1	f	cattggcttgcgagacgtagac
	g	gctgaaggtctcttccatcacc
Fizz-1	f	caaggaacttcttgccaatccag
	g	ccaagatccacaggcaaagcca
Abca1	f	ggagcctttgtggaactcttcc
	b	cgctctcttcagccactttgag
Abcg1	f	gacaccgatgtgaacccgtttc
	b	gcatgatgctgaggaaggtcct
Klf4	f	ctatgcaggctgtggcaaaacc
	b	ttgcggtagtgcctggtcagtt
LXRα	f	atcgccttgctgaagacctctg
	b	ctgctttggcaaagtcttcccg
RXRα	f	gtgaaagatgggattctcctggc
	b	gtcacgcatcttagacaccagc
Irf7	f	gagactggctattgggggag
	g	gaccgaaatgcttccaggg
Irf8	f	caatcaggaggtggatgcttcc
	g	gttcagagcacagcgtaacctc
C/ebpβ	f	caacctggagacgcagcacaag
	g	gcttgaacaagttccgcagggt
Nos2	f	gagacagggaagtctgaagcac
	b	ccagcagtagttgctcctcttc
Cox-2	f	gcgacatactcaagcaggagca
	b	agtggtaaccgctcaggtgttg

Human Gene	Direction	Sequence (5’ → 3’)
GAPDH	f	gtctcctctgacttcaacagcg
	g	accaccctgttgctgtagccaa
mouse/human MafB	f	gacgcagctcattcagcag
	b	ccggagttggcgagtttct
c-MAF	f	gcaatgagcaactccgacctg
	b	gccggtcatccagtagtagtct
MRC1 (CD206)	f	agccaacaccagctcctcaaga
	b	caaaacgctcgcgcattgtcca

ABCA1	f	caggctactacctgaccttggt
	b	ctgctctgagaaacactgtcctc
ABCG1	f	gagggatttgggtctgaactgc
	b	tctcaccagccgactgttctga
KLF4	f	catctcaaggcacacctgcgaa
	b	tcggtcgcatttttggcactgg
LXRα	f	tggacacctacatgcgtcgcaa
	b	caaggatgtggcatgagcctgt
RXRα	f	ttgccaagcagccgacaaacag
	b	aaggaggcgatgagcagctcat
IRF8	f	aggtcttcgacaccagccagtt
	b	gcacgagaatgagtttggagcg
C/EBPβ	f	agaagaccgtggacaagcacag
	b	ctccaggaccttgtgctgcgt

Supplementary Table S2. Real-time PCR Primers.

Probe	Direction	Sequence (5’ → 3’)
Human FASN (LXRE)	f	tggacgtccgtctcgggtctg
	b	cgcaagtgcgggcgttgacc
Mouse Fasn (SRE, LXRE)	f	cgcagccccgacgctcattg
	b	cgccggcgctatttaaaccgc
Human ABCA1 (LXRE)	f	atgtgtcgtgggcggctgaac
	b	tatagattcggctgcaccgagc
Mouse Abca1 (LXRE)	f	ctacataaacagaggccgggaag
	b	atagattcggctgtgccgagc
Mouse Arg1 (AP1)	f	tagacagtgtaacctggtgaca
	b	gcaaggtgaggctaaaacagc
Human MafB (Stat6, C/ebp)	f	cctttgcgcacagtggccact
	b	gctccgagtagctctccactg

Supplementary Table S3. Chromatin immunoprecipitation RT-PCR Primers.

References

1 Ramsey, S. A. *et al.* Epigenome-guided analysis of the transcriptome of plaque macrophages during atherosclerosis regression reveals activation of the Wnt signaling pathway. *PLoS genetics* **10**, e1004828, (2014).

2 Rayner, K. J. *et al.* Inhibition of miR-33a/b in non-human primates raises plasma HDL and lowers VLDL triglycerides. *Nature* **478**, 404-407, (2011).

3 Rottiers, V. *et al.* Pharmacological inhibition of a microRNA family in nonhuman primates by a seed-targeting 8-mer antimiR. *Science translational medicine* **5**, 212ra162, (2013).
